# Carcinome sébacé de la glande parotide

**DOI:** 10.11604/pamj.2015.21.132.7166

**Published:** 2015-06-17

**Authors:** Madiha Mahfoudhi, Khaled Khamassi

**Affiliations:** 1 Service de Médecine Interne A, Hôpital Charles Nicolle, Tunis, Tunisie; 2 Service ORL, Hôpital Charles Nicolle, Tunis, Tunisie

**Keywords:** Carcinome sébacé, glande parotide, évolution, Sebaceous carcinoma, parotide gland, evolution

## Image en medicine

Le carcinome sébacé primaire de la glande parotide est extrêmement rare. Il peur simuler d'autres maladies néoplasiques. Le tableau clinico-radiologique est variable. Seul l'examen anatomopathologique permet de confirmer le diagnostic. Un patient âgé de 54 ans hospitalisé pour exploration d'une tuméfaction indolore infra-auriculaire droite évoluant depuis 2 mois. L'anamnèse n'a pas noté de fièvre, ni d'altération de l’état général. L'examen physique a retrouvé une masse ferme indolore de 7 centimètres de grand axe au niveau de la loge parotidienne droite sans signes inflammatoires en regard. Il n'avait pas d'hépatomégalie ni de splénomégalie. Les aires ganglionnaires étaient libres. L'examen biologique a révélé un discret syndrome inflammatoire. L'IRM cervicale a montré une masse lobulaire intra-parotidienne droite avec un hyposignal en T1 et un hypersignal en T2 de la partie inférieure de la glande parotide droite. Elle n'a pas détecté d'adénopathies associées. Plusieurs diagnostics ont été évoqués en particulier un lymphome ou un liposarcome de la parotide. Il a subit une résection chirurgicale de la glande parotide droite. L'examen anatomo-pathologique a montré deux populations de cellules. Une population faite de cellules à petits noyaux arrondis avec un cytoplasme éosinophile et une autre correspondant à des cellules à cytoplasme vacuolaire avec des anomalies nucléaires associées. Le diagnostic d'un carcinome sébacé de la glande parotide droite a été retenu. La recherche d'une autre localisation tumorale était négative. Une radiothérapie de la loge parotidienne droite a été réalisée. L’évolution était marquée par l'absence de récidive tumorale avec un recul de 15 mois.

**Figure 1:**
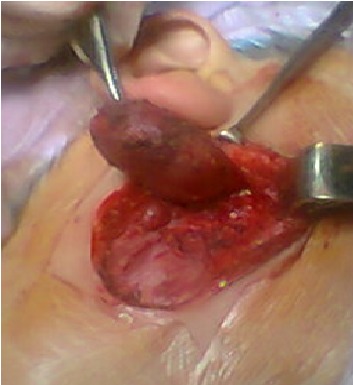
Pièce de résection chirurgicale de la glande parotide droite

